# Cross-Talk in the Female Rat Mammary Gland: Influence of Aryl Hydrocarbon Receptor on Estrogen Receptor Signaling

**DOI:** 10.1289/ehp.1509680

**Published:** 2015-09-15

**Authors:** Janina Helle, Manuela I. Bader, Annekathrin M. Keiler, Oliver Zierau, Günter Vollmer, Sridar V. Chittur, Martin Tenniswood, Georg Kretzschmar

**Affiliations:** 1 Institute of Zoology, Molecular Cell Physiology and Endocrinology, Technische Universität Dresden, Dresden, Germany; 2 Department of Biomedical Sciences, University at Albany, State University of New York, Albany, New York, USA

## Abstract

**Background:**

Cross-talk between the aryl hydrocarbon receptor (AHR) and the estrogen receptor (ER) plays a major role in signaling processes in female reproductive organs.

**Objectives:**

We investigated the influence of the AHR ligand 3-methylcholanthrene (3-MC) on ER-mediated signaling in mammary gland tissue of ovariectomized (ovx) rats.

**Methods:**

After 14 days of hormonal decline, ovx rats were treated for 3 days with 4 μg/kg 17β-estradiol (E2), 15 mg/kg 8-prenylnaringenin (8-PN), 15 mg/kg 3-MC, or a combination of these compounds (E2 + 3-MC, 8-PN + 3-MC). Whole-mount preparations of the mammary gland were used to count terminal end buds (TEBs). Protein expression studies (immunohistochemistry, immunofluorescence), a cDNA microarray, pathway analyses, and quantitative real-time polymerase chain reaction (qPCR) were performed to evaluate the interaction between AHR- and ER-mediated signaling pathways.

**Results:**

E2 treatment increased the number of TEBs and the levels of Ki-67 protein and progesterone receptor (PR); this treatment also changed the expression of 325 genes by more than 1.5-fold. Although 3-MC treatment alone had marginal impact on gene or protein expression, when rats were co-treated with 3-MC and E2, 3-MC strongly inhibited E2-induced TEB development, protein synthesis, and the expression of nearly half of E2-induced genes. This inhibitory effect of 3-MC was partially mirrored when 8-PN was used as an ER ligand. The anti-estrogenicity of ligand-activated AHR was at least partly due to decreased protein levels of ERα in ductal epithelial cells.

**Conclusion:**

Our data show transcriptome-wide anti-estrogenic properties of ligand-activated AHR on ER-mediated processes in the mammary gland, thereby contributing an explanation for the chemopreventive and endocrine-disrupting potential of AHR ligands.

**Citation:**

Helle J, Bader MI, Keiler AM, Zierau O, Vollmer G, Chittur SV, Tenniswood M, Kretzschmar G. 2016. Cross-talk in the female rat mammary gland: influence of aryl hydrocarbon receptor on estrogen receptor signaling. Environ Health Perspect 124:601–610; http://dx.doi.org/10.1289/ehp.1509680

## Introduction

To evaluate the influence of endocrine-disrupting natural compounds or selective estrogen receptor modulators, it is crucial to understand the underlying mechanisms. This study was designed to investigate the impact of ligand-activated aryl hydrocarbon receptor (AHR) on ligand-activated estrogen receptor (ER) signaling pathways in the rodent mammary gland.

The AHR is a ligand-dependent transcription factor that belongs to the basic helix-loop-helix/Per-Arnt-Sim (bHLH/PAS) family (Hankinson 1995; Matsumura and Vogel 2006; Mimura and Fujii-Kuriyama 2003; Poellinger 2000). The best-characterized ligands for the AHR are planar hydrophobic molecules, including many environmental contaminants such as halogenated aromatic hydrocarbons (HAHs) and polycyclic aromatic hydrocarbons (PAHs) (reviewed by Denison et al. 2002). After ligand binding, AHR translocates to the nucleus, dimerizes with its partner protein, aryl hydrocarbon receptor nuclear translocator (ARNT), and the AHR/ARNT-heterodimer binds to xenobiotic response elements (XREs), inducing gene expression for drug-metabolizing enzymes such as CYP1A1 and CYP1B1 (Beischlag et al. 2008; Larsen et al. 1998; Whitlock et al. 1996). In addition to its major role in xenobiotic metabolism, the AHR is involved in the regulation of cell proliferation, apoptosis, adipose differentiation, tumor suppressor functions, immune cell differentiation, and reproductive function (Hernández-Ochoa et al. 2009; Kerkvliet 2002; Puga et al. 2009). The physiological consequences of these actions on individual tissues have not yet been fully elucidated. In the mammary gland, the AHR is dispensable for development (Le Provost et al. 2002), but this receptor appears to be an important factor in the regulation of proliferation because in AHR-null mice, the number of terminal end buds (TEBs) is reduced by 50% during estrous-stimulated growth (Hushka et al. 1998). These AHR-mediated responses in the mammary gland are most likely due to cross-talk between AHR and steroid receptors, including ERα, ERβ, and androgen receptor (AR), as has been reported for cell lines and mouse uterus (Beischlag and Perdew 2005; Matthews et al. 2005; Ohtake et al. 2003, 2007, 2008). How ligand-activated AHR influences ER signaling on a molecular level remains unclear. Several different mechanisms have been proposed, including direct binding to ERs, competition between the AHR/ARNT complex and the ER for common cofactors (p300, p160, CBP), induction of CYPs by activated AHR leading to enhanced E2 metabolism, and proteasomal degradation of the ER triggered by liganded AHR (for an overview, see Shanle and Xu 2011; Swedenborg and Pongratz 2010).

PAHs are widespread AHR ligands that mainly form in the process of incomplete combustion and are therefore abundant constituents of exhaust fumes from cars and factories as well as of cigarette smoke. Because the synthetic PAH 3-methylcholanthrene (3-MC) has a carcinogenic potential that is higher than that of most other PAHs such as benzo[*a*]pyrene, it is frequently employed to induce tumor formation in mice (Chen et al. 2015; Rymaszewski et al. 2014). Furthermore, 3-MC is frequently used in studies investigating AHR signaling because it is known to be a strong activator of AHR (Riddick et al. 1994). In addition, 3-MC is readily metabolized, further decreasing its effectiveness regarding the activation of AHR *in vivo*, whereas other AHR ligands such as 2,3,7,8-tetrachlorodibenzo-*p*-dioxin (TCDD) are extremely persistent (Riddick et al. 1994). This characteristic makes 3-MC a suitable model to study effects of PAHs on ER signaling. In this paper, we describe the *in vivo* effects of E2, 3-MC, and E2 + 3-MC on transcriptional and translational regulation of mRNA and protein levels to develop a mechanistic understanding of the interactions between AHR- and ER-mediated signaling pathways in the rat mammary gland in a physiological context. We have used a transcriptome-based approach to characterize ER- and AHR-regulated genes and the overall expression change triggered by their respective receptors. Additionally, we treated rats with the potent phytoestrogen 8-prenylnaringenin (8-PN) (Milligan et al. 1999; Zierau et al. 2008) either alone or in co-treatment with 3-MC.

## Methods

*Substances.* 17β-Estradiol (E2) and 3-methylcholanthrene (3-MC) were purchased from Sigma-Aldrich. 8-Prenylnaringenin (8-PN) was synthesized from naringenin as described previously (Gester et al. 2001). The purity of the compound was assessed to be > 99% by gas chromatography and high-performance liquid chromatography (HPLC). 8-PN was used as a racemic mixture.

*Animals.* Young adult female Lewis rats (200 g) were maintained under controlled conditions of temperature (20 ± 1°C, relative humidity 50–80%) and illumination (12 hr light, 12 hr dark). Animals were housed in open cages (Tecniplast) with four to six animals per cage. A mixture of Lignocell wood chips and Rehofix corncob granules (JRS Rettenmaier & Söhne) was used for bedding. All rats had free access to standard phytoestrogen-free rat diet (Teklad Global Diet) and water. Animals used in this study were treated humanely and with regard for the alleviation of suffering. The experimental design complied with the ARRIVE guidelines published by the National Centre for the Replacement, Refinement and Reduction of Animals in Research (https://www.nc3rs.org.uk/). All procedures were approved by and carried out according to the institutional and state Animal Care and Use Committee guidelines mandated by the German federal law for animal welfare.

*Three-day* in vivo *assay.* Approximately 10-week-old rats were bilaterally ovariectomized (ovx). Fourteen days after ovx, rats were treated subcutaneously with the test compounds (E2, 3-MC, E2 + 3-MC, 8-PN, and 8-PN + 3-MC) in the same room in which the animals were housed. The animals were treated 2 hr after the start of the light period of the day; three treatments were administered at intervals of 24 hr. E2 was administered at 4 μg/kg body weight (BW)/day, and 3-MC and 8-PN were administered at 15 mg/kg BW/day. The combination doses for E2 + 3-MC were 4 μg/kg BW + 15 mg/kg BW, respectively, and the doses for 8-PN + 3-MC were 15 mg/kg BW + 15 mg/kg BW, respectively. The substances were dissolved in a dimethyl sulfoxide (DMSO)/castor oil mixture that also served as the negative control (hereafter referred to as vehicle). The animals were randomly selected for treatment and vehicle groups (E2 group, *n* = 13; vehicle group, *n* = 11; other treatment groups, *n* = 6). The animals were sacrificed by CO_2_ inhalation subsequent to a light O_2_/CO_2_ anesthesia 24 hr after the final treatment, and necropsies were performed. A whole mount of the 4th right mammary gland was prepared, and the 4th left mammary gland was excised and fixed in 4% paraformaldehyde before embedding in paraffin. The 2nd, 3rd, and 5th mammary glands from both sides were snap-frozen in liquid nitrogen for RNA preparation.

*RNA preparation.* Total cytoplasmic RNA was extracted from the mammary gland tissue by the standard TRIzol® method (Life Technologies). The quality and the concentrations of the preparations of total RNA were determined using a NanoDrop spectrophotometer (Thermo Fisher Scientific) and an Agilent Bioanalyzer (Agilent Technologies).

*mRNA microarray analyses.* Total RNA (100 ng) from three rats per treatment group (vehicle, E2, 3-MC, E2 + 3-MC) that was deemed to be of good quality [RNA integrity number (RIN) ≥ 7] was processed according to the standard Affymetrix Whole Transcript Sense Target labeling protocol. The fragmented biotin-labeled cDNA was hybridized for 16 hr to Affymetrix Gene 1.0 ST arrays and scanned on an Affymetrix Scanner 3000 7G using AGCC software (Affymetrix, Inc.). The resulting CEL files were analyzed for quality using Affymetrix Expression Console software and were then imported into GeneSpring GX11.5 (Agilent Technologies). The data were quantile-normalized using the Probe Logarithmic Intensity Error (PLIER) method and baseline-transformed to the median of the control samples. The probe sets were further filtered to exclude the bottom 20th percentile across all samples. The resulting entity list was subjected to an unpaired *t*-test with Benjamini–Hochberg false discovery rate correction and a 1.5-fold filter to identify differentially expressed transcripts between conditions at a *p*-value < 0.05. Pathway analysis and functional annotation clustering of E2-regulated genes (*p*-value < 0.05) were performed using Database for Annotation, Visualization and Integrated Discovery (DAVID) 6.7 (Huang et al. 2009). All data have been deposited in the Gene Expression Omnibus (GEO, accession number GSE64636; Edgar et al. 2002).

qPCR *validation of microarray data.* Changes in mRNA identified by microarray were validated using five to six independent biological replicates. Reverse transcription PCR reactions were performed with 1.5 μg total RNA using Taqman® Reverse Transcription Reagents (Applied Biosystems) to synthesize cDNA for mRNA expression analysis. qPCR analyses were performed according to a previously described protocol (Wang WL et al. 2011) and were analyzed as follows using the ABI 7900HT Fast Real-Time PCR System (Applied Biosystems): first denaturing cycle at 95°C for 3 min, then 95°C for 15 sec, 60°C for 10 sec, and 72°C for 20 sec, repeated for 40 cycles. Fluorescence was quantified at the end of the 72°C annealing step, and the amplicon specificity was confirmed by a melting curve analysis (60–95°C). Primer sequences are summarized in the Supplemental Material (Table S1). The relative mRNA amounts of target genes were calculated after normalization to an endogenous reference gene (ribosomal protein S18). The results were obtained using the 2^–ΔΔCT^ method (Pfaffl 2001) and are expressed as relative amounts of mRNA compared with those of the vehicle-treated animals.

*Mammary gland whole-mount preparations.* Whole-mount preparations were performed as previously described (de Assis et al. 2010). After staining with Carmine Alum (0.2% carmine, 0.5% aluminum potassium sulfate), the samples were coated with polyacrylate, placed in a water-filled petri dish, and viewed under a stereomicroscope (Stemi DRC, Carl Zeiss AG) to count the number of terminal end buds (TEBs). The TEBs of at least six animals from every treatment group were counted.

*Immunohistochemistry.* Dissected mammary tissue was fixed in 4% paraformaldehyde, embedded in paraffin, and sectioned at 3 μm. Antigen retrieval was performed with 10 mM Tris-EDTA, pH 9 at 60°C overnight. Proteins of interest were stained using the IHC Select^TM^ Immunoperoxidase Secondary Detection System (Millipore) according to the manufacturer’s protocol. Three primary antibodies [anti-progesterone receptor (PR) (1:350; Thermo Fisher Scientific), anti-Ki-67, a cell proliferation marker (1:150; Abcam), and anti-AHR (1:250; Abcam)] were diluted in 5% filtered skim milk powder in phosphate-buffered saline (PBS) and incubated for 1 hr at room temperature (18–20°C) in a humidified chamber. Sections were counterstained with hematoxylin (Carl Roth). The samples were visualized using a Keyence BZ-8100E (Keyence) microscope. To quantify Ki-67– and PR-positive cells, ≥ 500 luminal epithelial cells from each of five or more animals per group were analyzed and scored using ImageJ 1.47 (Schneider et al. 2012). AHR expression was quantified by measuring the 3,3´-diaminobenzidine (DAB) staining intensity of luminal epithelial cells normalized to that of the negative control group using ImageJ 1.47 (Schneider et al. 2012). For fluorescent ERα staining, tissue sections were blocked with 5% bovine serum albumin/tris-buffered saline (BSA/TBS) + 0.1% Triton X-100 for 1 hr at room temperature in a humidified chamber. Primary anti-ERα antibody (1:250; Abcam) was diluted in 2.5% BSA/TBS, and the tissue sections were incubated overnight at room temperature. After washing with TBS, sections were incubated with CruzFluor™ 488 conjugated goat-anti rabbit secondary antibody (1:200 in 2.5% BSA/TBS; Santa Cruz Biotechnology) for 1 hr. Sections were mounted with Mowiol® (Carl Roth) and counterstained with diamidino-2-phenylindole (DAPI; 1:250; Santa Cruz). An Axiovert 100 microscope with a Colibri.2 LED illumination system (Carl Zeiss AG) was used to visualize the fluorescence-stained mammary gland epithelial cells. Magnification and exposure time were set identically to identify fluorescence intensity differences between treatment groups. The fluorescence intensity per duct was measured and normalized to that of the negative control group using ImageJ 1.47 (Schneider et al. 2012).

*Statistical analysis.* The data are presented as the arithmetic mean ± standard deviation (SD) for immunohistochemistry and qPCR data or as the median with percentiles (25–75%) for TEB count and immunofluorescence data. Statistical analysis included one-way analysis of variance (ANOVA) followed by the Bonferroni post hoc test to determine significant differences for the qPCR results, TEB count, immunohistochemistry, and immunofluorescence in the mammary gland. The results were considered to be statistically significant at *p* ≤ 0.05.

## Results

The affinity of 3-MC for the AHR is somewhat lower than that of the prototypical ligand TCDD. The half-maximal inhibitory concentration (IC_50_) of 3-MC in a competitive ligand-binding assay is ~4-fold higher than that of TCDD, and the concentration of 3-MC necessary to induce *Cyp1a1* mRNA expression is between 10- and 100-fold higher (Riddick et al. 1994). We therefore opted for a much higher treatment dose of 15 mg 3-MC per kg body weight per day than the dose typically used for treatment with TCDD. This dose is similar to that used in many other studies of 3-MC as an AHR ligand (Deb and Bandiera 2010; Göttlicher and Cikryt 1987; Kondraganti et al. 2005).

**Figure 1:**
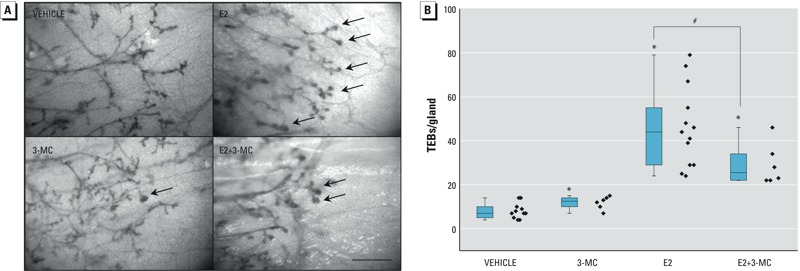
Effects of 17β-estradiol (E2) and 3-methylcholanthrene (3-MC), alone and in combination, on terminal end bud (TEB) formation in ovariectomized (ovx) rats. Ovx rats were treated with E2, 3-MC, E2 + 3-MC, or vehicle control for 3 days. Mammary gland whole mounts were prepared and stained as described in “Methods.” (*A*) Representative TEB formation in mammary whole mounts from each treatment group. Arrows highlight TEBs. Magnification scale: 500 μm. (*B*) Quantification of the effects of E2 and 3-MC, alone and in combination, on TEB formation. Diamonds represent individual animals; boxes indicate 25th to 75th percentiles, and the horizontal lines indicate the median. The whiskers indicate the highest and the lowest measured values partitioned by the median. *Significant differences (*p* < 0.05) from vehicle control [one-way analysis of variance (ANOVA)]. #Significant differences (*p* < 0.05) from E2 treatment alone (one-way ANOVA).

*Effects of 3-MC on terminal end bud development.* To determine the nature of the interaction between the ligand-activated AHR and ER pathways, we assessed the effects of treating ovx rats for 3 days with E2 and 3-MC, alone or in combination, on the number of TEBs in whole-mount preparations of the mammary gland (Figure 1A). Quantitation of these data is presented in Figure 1B. The number of TEBs was significantly increased in estradiol-treated rats (46.2 ± 18.2) compared with vehicle-treated controls (8.1 ± 3.5). Differences in TEB numbers were also significant between 3-MC– (11.8 ± 2.9) and vehicle-treated controls, albeit to a far lesser extent. Co-treatment with E2 and 3-MC significantly reduced the number of TEBs compared with treatment with E2 alone (29.2 ± 9.5), although the number of TEBs remained higher than in the 3-MC–treated animals. These data suggest that the liganded AHR pathway attenuates, but does not completely block, E2 signaling mediated by the ER.

**Figure 2:**
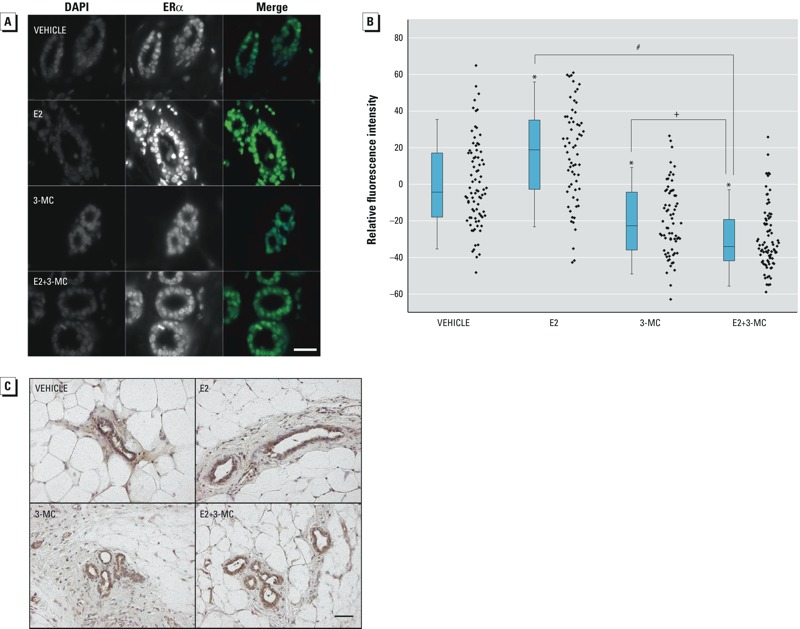
Effects of 17β-estradiol (E2) and 3-methylcholanthrene (3-MC), alone and in combination, on estrogen receptor alpha (ERα) and aryl hydrocarbon receptor (AHR) status in mammary glands from ovariectomized (ovx) rats. Ovx rats were treated with E2, 3-MC, E2 + 3-MC, or vehicle control for 3 days. (*A*) Representative expression of ERα in mammary glands from each treatment group. Tissue sections were prepared for immunofluorescence microscopy as described in “Methods.” Magnification scale: 30 μm. DAPI, diamidino-2-phenylindole. (*B*) Quantification of the effects of E2 and 3-MC, alone and in combination, on ERα expression in the mammary glands of 6 animals per treatment group. Fluorescence intensity per duct was measured as described in “Methods” and normalized to the vehicle control. Diamonds represent individual ducts; boxes show the 25th and 75th percentiles, and the horizontal lines indicate the median. The whiskers mark the standard deviation for each treatment group. (*C*) Representative immunohistochemical staining for AHR in mammary gland ducts labeled with an antibody against AHR for each treatment group. Paraffin-embedded sections were prepared for immunohistochemistry using 3,3’-diaminobenzidine (DAB) as described in “Methods.” Magnification scale: 50 μm. *Significant differences (*p* < 0.05) from vehicle control [one-way analysis of variance (ANOVA)]. #Significant differences (*p* < 0.05) from E2 treatment alone (one-way ANOVA). +Significant differences (*p* < 0.05) from 3-MC treatment alone (one-way ANOVA).

*Protein levels of ER*α *and AHR.* To determine whether the effect of 3-MC on E2 stimulation of TEB formation was the result of changing receptor levels, we assessed the status of ERα and AHR in the mammary gland. The relative mRNA levels of the ER (*Esr1, Esr2*) and of *Ahr* were not significantly different in any of the treatment groups as measured by qPCR (see Supplemental Material, Figure S1). The steady-state protein levels of ERα were significantly increased in the ductal epithelial cells after treatment with E2 alone compared with levels in the vehicle control group (Figure 2A,B). In contrast, 3-MC alone caused a significant decrease in the relative fluorescence intensity compared with that of the vehicle control, and 3-MC further abrogated the effect of E2 on ERα expression when co-administered (Figure 2B). This finding suggests that 3-MC exerts a strong anti-estrogenic effect by down-regulating ERα expression. In contrast, neither 3-MC nor E2 induced any significant changes in the AHR protein in ductal epithelial cells of the mammary glands, either alone or in combination (Figure 2C; vehicle, 100 ± 17.7% relative fluorescence intensity; E2 98.9 ± 10.1% relative fluorescence intensity; 3-MC 96.8 ± 15.2% relative fluorescence intensity; E2 + 3-MC 99.3 ± 7.4% relative fluorescence intensity).

**Figure 3:**
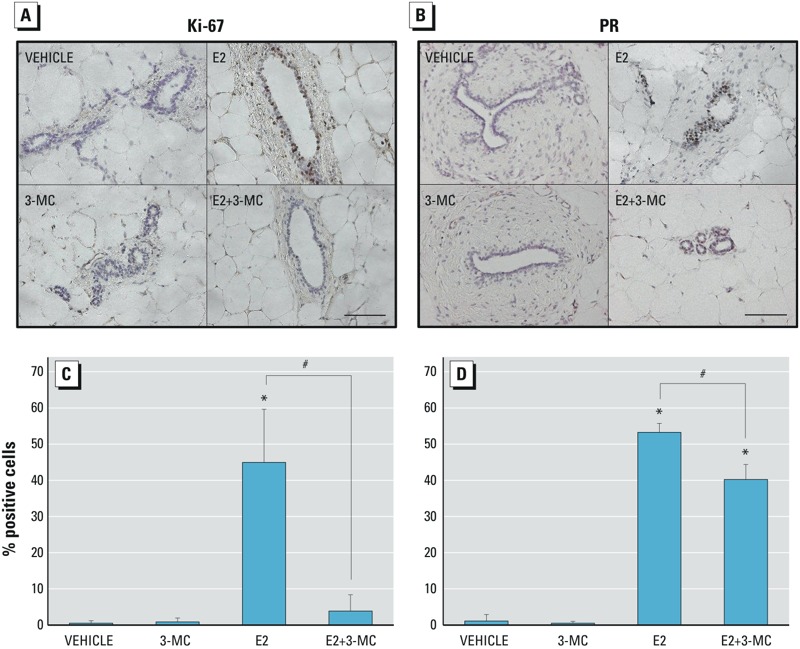
Ki-67 and progesterone receptor (PR) protein expression in the mammary gland. Ovariectomized (ovx) rats were treated with 17β-estradiol (E2), 3-methylcholanthrene (3-MC), E2 + 3-MC, or vehicle control for 3 days. Paraffin-embedded sections were prepared for immunohistochemistry using 3,3’-diaminobenzidine (DAB) as described in “Methods.” Paraffin-embedded sections of rat mammary glands from each treatment group were stained with DAB using antibodies against Ki-67 (*A*) or PR (*B*) and counterstained with hematoxylin. Magnification scale: 50 μm. (*C*,*D*) Quantification of the effects of E2 and 3-MC, alone and in combination, on Ki-67 and PR protein levels. The number of positively stained epithelial cells were counted and normalized to the total number of epithelial cells; ≥ 500 cells were evaluated per animal, and five to six animals were used per treatment group. Data are presented as the arithmetic mean ± SD. *Significant differences (*p* < 0.05) from vehicle control [one-way analysis of variance (ANOVA)]. #Significant differences (*p* < 0.05) from E2 treatment alone (one-way ANOVA).

*Effects of 3-MC on* Ki-67 *and* PR *expression.* To investigate the cellular mechanisms responsible for the anti-estrogenic effects of 3-MC, we examined the expression of the proliferation marker *Ki-67* and of *PR*, a classical E2-regulated target in the mammary ductal epithelium (Haslam and Shyamala 1979). As shown in Figure 3A,C, the percentage of ductal epithelial cells expressing *Ki-67* increased from 1% in the vehicle-treated control animals to 44.9% after 3 days of E2 treatment. 3-MC alone did not increase *Ki-67* expression, but it completely attenuated E2-induced expression of *Ki-67* (4%). Similarly, treatment with E2 increased the number of PR-positive epithelial ductal cells to 53%. However, although co-administration of 3-MC significantly reduced PR-positive epithelial ductal cells to 40% (Figure 3D), it did not completely abrogate the E2-induced increase in PR levels. These data suggest that although many of the effects of 3-MC on E2 regulation of tissue growth may be attributable to its effects on ER levels, some of the physiological effects of ER are more sensitive to 3-MC than others.

*Genome-wide transcriptome analysis.* The effects of E2, 3-MC, and E2 + 3-MC on gene expression in the mammary gland were assessed by cDNA microarray. A 1.5-fold change filter was used to compare the changes in expression resulting from the respective treatments (3-MC, E2, E2 + 3-MC) to the vehicle controls.

**Figure 4:**
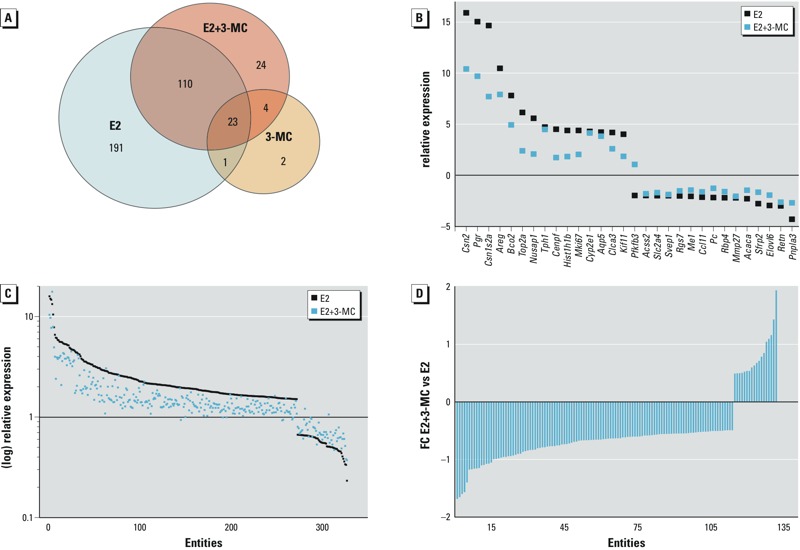
Genome-wide transcriptome analysis of the effects of 17β-estradiol (E2) and 3-methylcholanthrene (3-MC), alone and in combination, in mammary glands from ovariectomized (ovx) rats. Ovx rats were treated for 3 days with E2, 3-MC, E2 + 3-MC, or vehicle control. RNA was extracted from mammary glands from each treatment group, and global gene expression changes were analyzed by microarray as described in "Methods." Three independent biological replicates were interrogated for each treatment group. (*A*) Venn diagram illustrating the number of entities modulated by each treatment and overlap in target genes between treatment groups. Fold change (FC) ≥ 1.5 compared with the vehicle control. (*B*) FC of all entities modulated by entities from the mammary gland of E2 or E2 + 3-MC treated rats compared with vehicle-treated control rats. (*C*) Comparison of the FC of the 15 most up- and down-regulated genes by E2 and E2 + 3-MC treatment. (*D*) Interaction analysis for E2- and 3-MC–mediated gene expression. The FC of entities regulated by E2 + 3-MC and E2 are compared. The relative expression values of the E2 treatment group were set to 0. FC ≥ 1.5 compared with the E2 group.

Treatment with E2 differentially modulated the expression of 325 entities (Figure 4A), including 272 entities (84%) that were up-regulated and 54 (17%) that were down-regulated compared with the vehicle-treated controls. The expression change of the 15 genes that were most up-regulated by E2 ranged from 16-fold for beta-casein (*Csn2*) to 4-fold for kinesin family member 11 (*Kif11*) (Figure 4B). Although a number of genes were down-regulated by E2, the magnitude of the down-regulation was less dramatic, ranging from –4- to –2-fold for the 15 most–down-regulated genes. Pathway analyses of E2-regulated genes identified five predominant pathways [cell cycle, cell adhesion molecules, the p53 signaling pathway, pyruvate metabolism, and the insulin signaling pathway, *p* ≤ 0.05 (see Supplemental Material, Table S2)]. Genes involved in the control of cell division were exclusively up-regulated by E2 (see Supplemental Material, Table S2). The regulatory role of these E2 up-regulated genes in proliferative processes was confirmed by functional annotation clustering (see Supplemental Material, Figure S2). 3-MC alone modulated a total of 30 differentially expressed entities (Figure 4A), of which 25 were up-regulated and 5 were down-regulated. However, only 2 of these entities (*Lefty1* and the mRNA for the uncharacterized protein LOC502684) were solely regulated by 3-MC, both of which were close to the 1.5 cut-off point, indicating that under these experimental conditions, 3-MC alone had minimal effects on mammary gland gene expression. Moreover, 3-MC substantially attenuated E2-mediated signaling (Figure 4C) and dampened the inductive and suppressive effects of E2 on both up-regulated and down-regulated expression of 132 of the 325 genes modulated by E2 (Figure 4D). These data show that 3-MC has a significant global antagonist action on the biological activity of E2 in the mammary gland of ovx rats.

**Figure 5:**
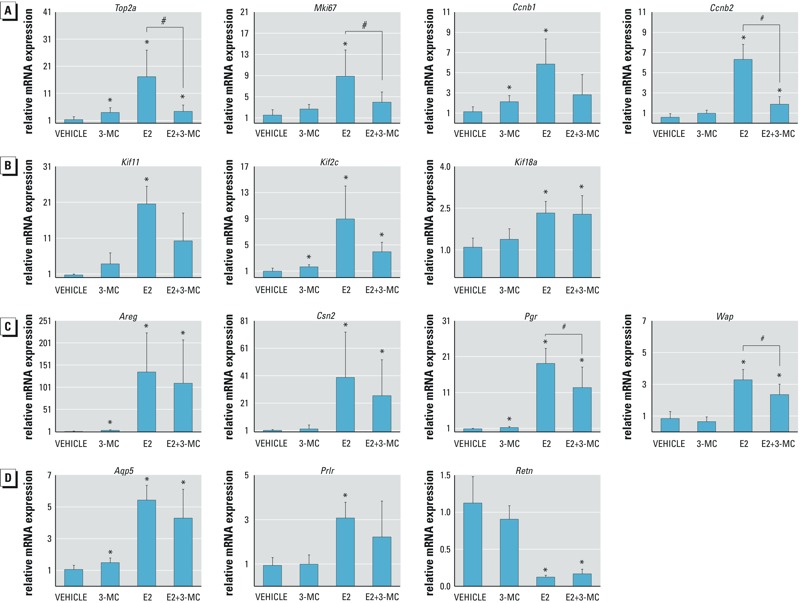
Expression of selected genes in mammary glands in ovariectomized (ovx) rats in response to 17β-estradiol (E2) and 3-methylcholanthrene (3-MC), alone and in combination. Ovx rats were treated for 3 days with E2, 3-MC, E2 + 3-MC, or vehicle control. RNA was extracted from the mammary glands, and expression of individual genes was assessed using quantitative polymerase chain reaction (qPCR)**as described in “Methods.” (*A*) Genes associated with cell cycle and proliferation; (*B*) genes associated with cytokinesis; (*C*) classical E2-responsive genes; (*D*) genes associated with differentiated mammary gland function. Data are presented as fold change, mean ± SD, for each treatment group relative to vehicle-treated controls. Five to six animals per treatment group were analyzed. *Significant differences (*p* < 0.05) from vehicle control [one-way analysis of variance (ANOVA)]. #Significant differences (*p* < 0.05) from E2 treatment alone (one-way ANOVA).

*qPCR validation of selected genes.* To validate the regulation of differentially regulated genes identified in the microarray, selected genes associated with the major ontologies affected by E2 (cell cycle, cell adhesion, and p53-mediated signaling) were analyzed using qPCR. E2 had a significant effect on the expression of genes associated with cell division (*Top2a*, *Mki67*, *Ccnb1*, *Ccnb2*; Figure 5A) and cytokinesis (*Kif11*, *Kif2c*, *Kif18a*; Figure 5B). Although treatment with 3-MC alone had an effect on levels of *Top2a*, *Ccnb1*, and *Kif2c* mRNA that was far less pronounced than for treatment with E2 alone, 3-MC significantly attenuated the up-regulation of *Top2a*, *Ccnb2*, and *Mki67* by E2. We also studied the effects of E2 and 3-MC on the expression of several E2-regulated genes responsible for differentiated functions of the mammary gland, including genes associated with the synthesis and secretion of milk (*Areg*, *Csn2*, *Pgr*, *Wap*, *Aqp5*, and *Prlr*), which were shown to be up-regulated by E2 in the microarray, and *Retn,* which was suppressed by E2 (Figure 5C,D). Although 3-MC alone had a small but statistically significant effect on *Areg*, *Pgr*, and *Aqp5* expression, it significantly reduced the up-regulation of *Pgr* and *Wap* by E2.

**Figure 6:**
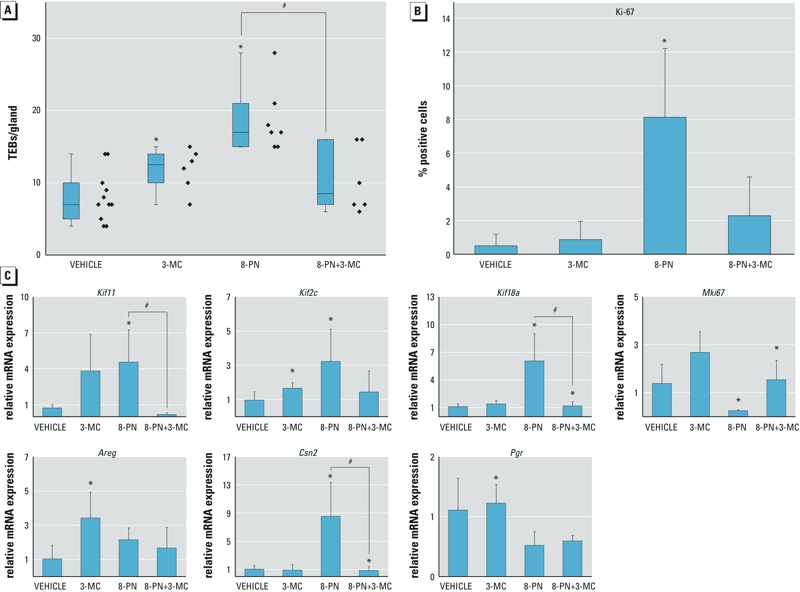
Effect of 8-prenylnaringenin (8-PN) and 3-methylcholanthrene (3-MC), alone and in combination, on terminal end bud (TEB) number, *Ki-67* staining, and mRNA expression in mammary glands from ovariectomized (ovx) rats. Ovx rats were treated for 48 hr with 3-MC, 8-PN, 8-PN + 3-MC, or vehicle control as described in “Methods.” (*A*) Quantitation of the effects of 8-PN and 3-MC, alone and in combination, on TEB formation in mammary whole mounts. Mammary gland whole mounts were prepared, stained, and quantitated as described in “Methods.” Diamonds represent individual animals; boxes indicate the 25th and 75th percentiles, and the horizontal lines indicate the median. The whiskers indicate the highest and the lowest measured values for each treatment group. (*B*) *Ki-67* expression in mammary glands from ovx rats treated with 8-PN or 3-MC alone or in combination. Paraffin-embedded sections of rat mammary glands from each treatment group were stained with 3,3’-diaminobenzidine (DAB) using antibodies against *Ki-67* and were counterstained with hematoxylin. The number of positively stained epithelial cells were counted and normalized to the total number of epithelial cells; ≥ 500 cells were evaluated per animal, and five to six animals were used per treatment group. Data are presented as the arithmetic mean ± SD. (*C*) Quantitative polymerase chain reaction (qPCR) analysis of selected genes in mammary glands from ovx rats treated with 8-PN or 3-MC alone or in combination. The results are presented as the mean ± SD, *n* = 5–6. *Significant differences (*p* < 0.05) from vehicle control [one-way analysis of variance (ANOVA)]. #Significant differences (*p* < 0.05) from 8-PN treatment alone (one-way ANOVA).

*Influence of 3-MC on 8-PN–induced effects.* To determine whether the effects of 3-MC are ER-ligand dependent and whether the abrogation of estrogenic signaling by 3-MC is important in natural-compound endocrine-disruptor interactions, rats were treated with the phytoestrogen 8-PN alone or in combination with 3-MC, recapitulating the experiments described above. 8-PN significantly increased the number of TEBs, although it was clear that in this bioassay system, 8-PN was significantly less estrogenic than E2, inducing 18.7 ± 4.6 versus 46.2 ± 18.2 TEBs per gland (Figure 1B and Figure 6A). The inductive effect of 8-PN was inhibited by 50% by co-treatment with 3-MC (Figure 6A). The increase in TEBs in response to 8-PN was reflected in the increase in the percentage of *Ki-67*–positive cells, which was substantially lower than with E2 treatment (8.1 ± 4.1% vs. 44.9 ± 14.7%, respectively), confirming the weaker estrogenic potency of 8-PN. Co-treatment with 3-MC effectively blocked the 8-PN–induced increase in proliferating cells, but statistical significance was not achieved (*p* = 0.05891, Figure 6B). To investigate the effects on gene expression of 8-PN alone and in co-treatment with 3-MC, we chose genes that were identified in earlier experiments as being highly regulated by E2 (*Areg, Csn2, Pgr*) as well as genes associated with proliferation (*Kif*, *Mki67*; Figure 6C). Although 8-PN weakly induced the expression of some of the E2 target genes (*Kif11*, *Kif2c*, *Kif18a*, *Csn2*), a significant counteracting effect of 3-MC on 8-PN–induced gene expression was observed for *Csn2, Kif18a*, and *Kif11*, in which the relative expression of the mRNA was significantly abrogated after co-treatment. However, some of the gene expression patterns induced by 8-PN were different from those induced by E2 because 8-PN decreased *Mki67* expression and had no significant effects on *Areg* or *Pgr* expression.

## Discussion

Interaction of xenobiotics with the hormonal system in the mammary gland is of great relevance regarding cancer risks and other syndromes whose etiologies are associated with hormonal interferences. In the present study, we investigated the cross-talk between ER- and AHR-mediated signaling processes in the female rat mammary gland using an ovariectomized rat mammary gland model system. This is a very sensitive model system for examining the physiological effects of E2 and the interactions between E2 and environmental AHR ligands such as TCDD and 3-MC.

The data presented herein are in line with the large body of literature demonstrating antiestrogenic effects of AHR ligands in cell culture (Gierthy et al. 1987; Safe and Krishnan 1995; Ueng et al. 2004; Wang F et al. 1998; Wormke et al. 2000). We demonstrated that 3-MC has inhibitory effects on the expression of a significant proportion of E2-modulated genes and has pronounced effects on physiological responses to E2. Within the last 15 years, there have been several reports of the inhibitory effects of TCDD on mammary gland development in both animals (Fenton et al. 2002; Vorderstrasse et al. 2004) and humans (Den Hond et al. 2002). In addition, animal studies (Collins et al. 2009; Vorderstrasse et al. 2004) and studies using primary mammary epithelial cells and HC-11 cells (Basham et al. 2015) suggested that AHR activation has a negative impact on milk production, resulting in the inability of mice to nutritionally support their offspring (Vorderstrasse et al. 2004). The inhibition of E2-induced development of TEBs, cell proliferation, up-regulation of the cell cycle, and proliferation-related pathways that we observed in response to 3-MC treatment suggests that these effects are independent of the specific AHR ligand and could therefore also be caused by the much more abundant PAHs. Furthermore, our data suggest that the adverse effects of AHR ligands on mammary gland development are most likely based on their ER-antagonistic properties. These data also offer an explanation for the observed decrease of mammary tumors in female rats after long-term treatment with TCDD (Kociba et al. 1978) and for the well-known inhibitory properties of exogenous AHR ligands on rodent mammary gland tumors and metastasis development (Lubet et al. 2011; Safe 2001; Wang T et al. 2011). Given the similarity between the development and carcinogenesis of rodent and human mammary glands (Rudel et al. 2011), the anti-estrogenic effects of AHR-mediated signaling may at least partially explain the increased overall survival and distant metastasis–free survival in hormone-dependent ER-positive breast cancers in human patients with elevated AHR expression (O’Donnell et al. 2014; Saito et al. 2013).

There are several mechanisms that might explain these ER-antagonistic effects. Many AHR ligands, among them 3-MC, have been shown to directly interact with ERs (Abdelrahim et al. 2006; Liu et al. 2006). In the case of 3-MC, this interaction is very weak but measurable. This affinity for both ERs may lead to activation of ERs in some tissues but to antagonistic effects in others. Notably, in mammary gland–derived HC11 cells, 3-MC acts as an AHR-independent ERα antagonist (Swedenborg et al. 2008), and this is therefore also likely to be the case in the mammary gland. In contrast, TCDD displays similar anti-estrogenic properties, although its affinity for ERα and ERβ is even lower and it is typically tested at much lower concentrations than 3-MC owing to its persistency. In the present study, only the expression changes of a subset of E2-regulated genes were blocked by 3-MC treatment. It is therefore likely that additional mechanisms are involved in mediating the anti-estrogenic response of AHR ligands in general and of 3-MC in particular. Firstly, AHR ligands alter hepatic E2 metabolism in female rats through the activation of several hepatic P450 cytochromes, increasing conversion of E2 to 2-hydroxyestradiol and 7α-hydroxyestradiol and thereby decreasing its bioavailability (Suchar et al. 1996). Secondly, the AHR has been identified as a component of a cullin 4B ubiquitin ligase complex (CUL4B^AHR^) (Ohtake et al. 2007), and in the rat uterus, activation of the AHR by 3-MC and other ligands of the receptor results in ubiquitination and proteasomal degradation of the ER and other steroid receptors (Wormke et al. 2003). A decrease in the level of the ligand (due to metabolism) and/or the receptor (due to degradation) would be anticipated to have global effects on E2-dependent gene expression in the mammary gland. Although 3-MC had significant inhibitory effects on E2-mediated modulation of many genes in the mammary gland (132/325, 41%), a great proportion (193/325, 59%) of the E2-responsive genes were not affected by 3-MC (Figure 4). This finding indicates that the effects of 3-MC on mammary gland physiology and gene expression cannot be solely (or even primarily) attributed to its effects on hepatic metabolism or on the stability of the estrogen receptor. This idea is also supported by the observed effects of 3-MC on the estrogenic actions of 8-PN, a naturally occurring, plant-derived ER ligand (Helle et al. 2014; Kitaoka et al. 1998; Milligan et al. 1999). Even though 8-PN is a weak estrogen and is metabolized differently in the liver (Guo et al. 2006; Keiler et al. 2015), the effects of 3-MC on 8-PN–mediated gene expression partially mirror effects observed with E2. Importantly, co-treatment with 3-MC also counteracted some of the effects of 8-PN, suggesting that the inhibitory effects of 3-MC are independent of which ligand is bound to the ER.

## Conclusions

Our data give a detailed account of the *in vivo* effects of E2 and 3-MC on gene expression and on development of mammary glands, and they demonstrate that AHR ligands such as 3-MC are anti-estrogenic with regard to many E2-regulated genes in the mammary gland. Almost no E2-independent effects of 3-MC on gene expression were observed, indicating that interaction with ERs is the predominant mode of action of AHR in the mammary gland. This finding helps to explain the adverse effects of AHR ligands on mammary gland development and underlines the potential of AHR as a breast cancer therapy target. We identified degradation of ERα as another putative mechanism by which 3-MC and possibly other PAHs inhibit ER signaling *in vivo*. Moreover, changes in gene expression caused by the phytoestrogen 8-PN were also reduced following 3-MC treatment, but further studies will be required to assess the importance of the interactions between ERs and AHR with regard to different endogenous, exogenous, nutrition-derived, and synthetic ER ligands *in vivo*.

## Supplemental Material

(550 KB) PDF
