# Supplementary material for: Cross-Talk in the Female Rat Mammary Gland: Influence of Aryl Hydrocarbon Receptor on Estrogen Receptor Signaling
Source: Environ Health Perspect. 2015 Sep 15;124(5):601–10. doi: 10.1289/ehp.1509680 (PMC4858405; doi:10.1289/ehp.1509680)
Supplement: (550 KB) PDF [file ehp.1509680.s001.acco.pdf]

Environ Health Perspect
DOI: 10.1289/ehp.1509680

Note to Readers: *EHP* strives to ensure that all journal content is accessible to all readers. However, some figures and Supplemental Material published in *EHP* articles may not conform to 508 standards due to the complexity of the information being presented. If you need assistance accessing journal content, please contact ehp508@niehs.nih.gov. Our staff will work with you to assess and meet your accessibility needs within 3 working days.

Supplemental Material

Cross-Talk in the Female Rat Mammary Gland: Influence of Aryl Hydrocarbon Receptor on Estrogen Receptor Signaling

Janina Helle, Manuela I. Bader, Annekathrin M. Keiler, Oliver Zierau, Günter Vollmer, Sridar V. Chittur, Martin Tenniswood, and Georg Kretzschmar

Table of Contents

Table S1. *qPCR* Primer sequences

Figure S1. Expression of *Esr1, Esr2* and *Ahr* in mammary glands in ovariectomized (ovx) rats in response to E2 and 3-MC, alone and in combination. Ovx rats were treated for three days with E2, 3-MC, E2+3-MC or vehicle control as described in methods. RNA was extracted from mammary glands as described in methods. Expression of individual genes was assessed using *qPCR* as described in methods. Data are presented as fold change, mean ± SD for each treatment group relative to vehicle treated controls. Five to six animals per treatment group were analyzed.

Table S2. Pathway analysis of E2-regulated genes in the mammary gland of ovx rats.

Figure S2. Functional clustering of E2-upregulated genes in the mammary gland. Functional annotation clustering was performed to assign E2-upregulated genes to associated biological

processes (GO= gene ontology). P-value associated with each annotation (GO) term ≥ 0.01 as determined by EASE Score (modified Fisher Exact p-value).

References

Table S1. *qPCR* Primer sequences.

gene	primer sequence
estrogen receptor α (*Esr1*)	Forward 5'-TGAAGCACAAGCGTCAGA GAGAT-3'<br>Reverse 5'-AGACCAGACCAATCATCAGGAT-3'
estrogen receptor β (*Esr2*)	Forward 5'-CTACAGAGAGATGGTCAAAAGTGGA-3'<br>Reverse 5'-GGGCAAGGAGACAGAAAGTAAGT-3'
aryl hydrocarbon receptor (*Ahr)*	Forward 5'-CCATGTCCATGTACCAGTGC-3'<br>Reverse 5'-TGAGCAGCAGTCTGAAGGTG-3'
ribosomal protein S18 *(Rps18)*	Forward 5'-CGTGAAGGATGGGAAGTATAGC-3'<br>Reverse 5'-TATTAACAGCAAAGGCCCAAAG-3'
casein beta (*Csn2*)	Forward 5'-CCCAAGCACAAACAGATGC-3'<br>Reverse 5'-GGGGCTGAGAAGAAACCAC-3'
progesterone receptor (*Pgr*)	Forward 5'-CTACTCGCTGTGCCTTACCA-3'<br>Reverse 5'-GGACCACCCCTTTCTGTCTT-3'
amphiregulin (*Areg*)	Forward 5'-CGGAAAAGGCAGAAGAAACA-3'<br>Reverse 5'-CTTACGGCGGAGACAAAGAC-3'
topoisomerase (DNA) II alpha (*Top2a*)	Forward 5'-CTGCCAAAGCCAAGAACAGT-3'<br>Reverse 5'-AAATCCCCTCACCCCTTAGA-3'
whey acidic protein (*Wap*)	Forward 5'-GCTTCATCAGCCTCGTTCTT-3'<br>Reverse 5'-CACACTCCTCGTTGGTTTGA-3'
prolactin receptor (*Prlr*)	Forward 5'-GCATCTTTCCACCAGTTCCT-3'<br>Reverse 5'-GCTCGTCCTCATTGTCATCA-3'
antigen identified by monoclonal antibody Ki-67 (*Mki67*)	Forward 5'-CAGTCCAGAACACCTAAAGCAA-3'<br>Reverse 5'-CAGGCTAATCTTGGGCAGAC-3'
cyclin B1 (*Ccnb1*)	Forward 5'-CCCTACCAAAACCTGTGGAC-3'<br>Reverse 5'-CATCGGAGAAAGCCTGACAC-3'
cyclin B2 (*Ccnb2*)	Forward 5'-TGGAGAGTGAAATACTGGAAGTCA-3'<br>Reverse 5'-TGAGAAGCACACGATGGAAG-3'
kinesin family member 11 *(Kif11*)	Forward 5'-GTGCGGATTGCTCTTCCA-3'<br>Reverse 5'-TCCTCCACTTTACCCTTCTCC-3'
kinesin family member 18A *(Kif18a*)	Forward 5'-AGCAGAACCGAGTGTAAAGAGG-3'<br>Reverse 5'-CCTTCGTTGGAAATGAGGAA-3'
kinesin family member 2C *(Kif2c*)	Forward 5'-CTGTCCTCCCAGATGTCCAG-3'<br>Reverse 5'-TCACGAAGGTCTCCAAGTCA-3'
aquaporin 5 (*Aqp5*)	Forward 5'-CTGCTCTTCCCCTCCTCTCT-3'<br>Reverse 5'-GGGTGCTTCAAACTCTTCGT-3'
resistin (*Retn*)	Forward 5'-AGTGCGGAAGCATAGACTGG-3'<br>Reverse 5'-ATCACCACCATCATCCCATT-3'

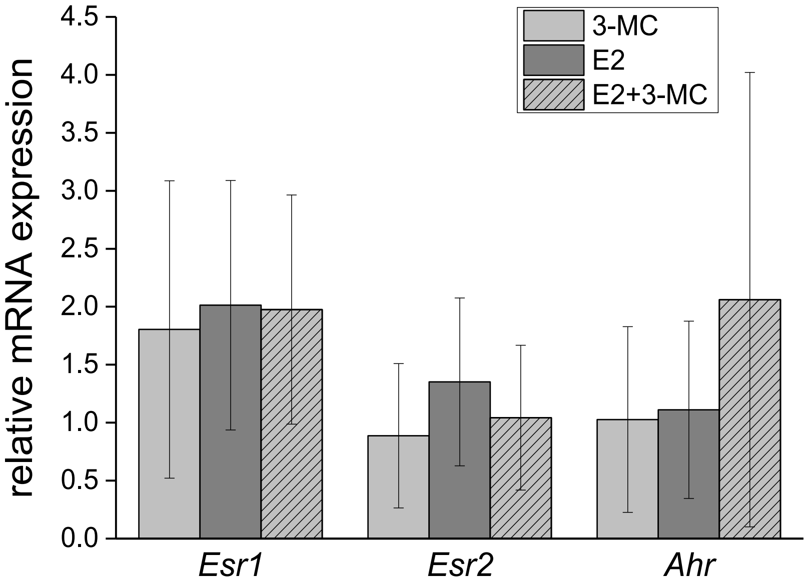

Figure S1. Expression of *Esr1, Esr2* and *Ahr* in mammary glands in ovariectomized (ovx) rats in response to E2 and 3-MC, alone and in combination.

Ovx rats were treated for three days with E2, 3-MC, E2+3-MC or vehicle control as described in methods. RNA was extracted from mammary glands as described in methods. Expression of individual genes was assessed using *qPCR* as described in methods.

Data are presented as fold change, mean ± SD for each treatment group relative to vehicle treated controls. Five to six animals per treatment group were analyzed.

Table S2. Pathway analysis of E2-regulated genes in the mammary gland of ovx rats.

Pathway[a]	E2 regulated genes involved[b]	p-value[c]
Cell cycle	↑ *Chek2, Ttk, Bub1, Bub1b, Cdc2, Ccnb2, Ccnb1, Mcm7, Pttg1, Plk1*	7.50E-05
Cell adhesion molecules (CAMs)	↑ *Cdh1, Cldn3, Cldn6, Cldn7, Cldn8, Glycam1, Mpzl1*<br>↓ *Pvrl3*	5.40E-03
p53 signaling pathway	↑ *Chek2, Cdc2, Ccnb1, Ccnb2, Serpinb5*	1.50E-02
Pyruvate metabolism	↓ *Acaca, Acss2, Me1, Pc*	1.80E-02
Insulin signaling pathway	↑ *Shc4, Ptpn1*<br>↓ *Acaca, Hk2, Pygl, Slc2a4*	4.20E-02

[a] Used pathways from the database Kyoto Encyclopedia of Genes and Genomes (KEGG) (Kanehisa and Goto 2000; Kanehisa et al. 2014).

[b] ↑↓ indicates relative expression values of genes up (↑) - or down (↓) - regulated by E2 compared to the vehicle control (fold change ≥ 1.5) as determined by a cDNA-microarray.

[c] Significance was determined by EASE Score, a modified Fisher Exact p-value.

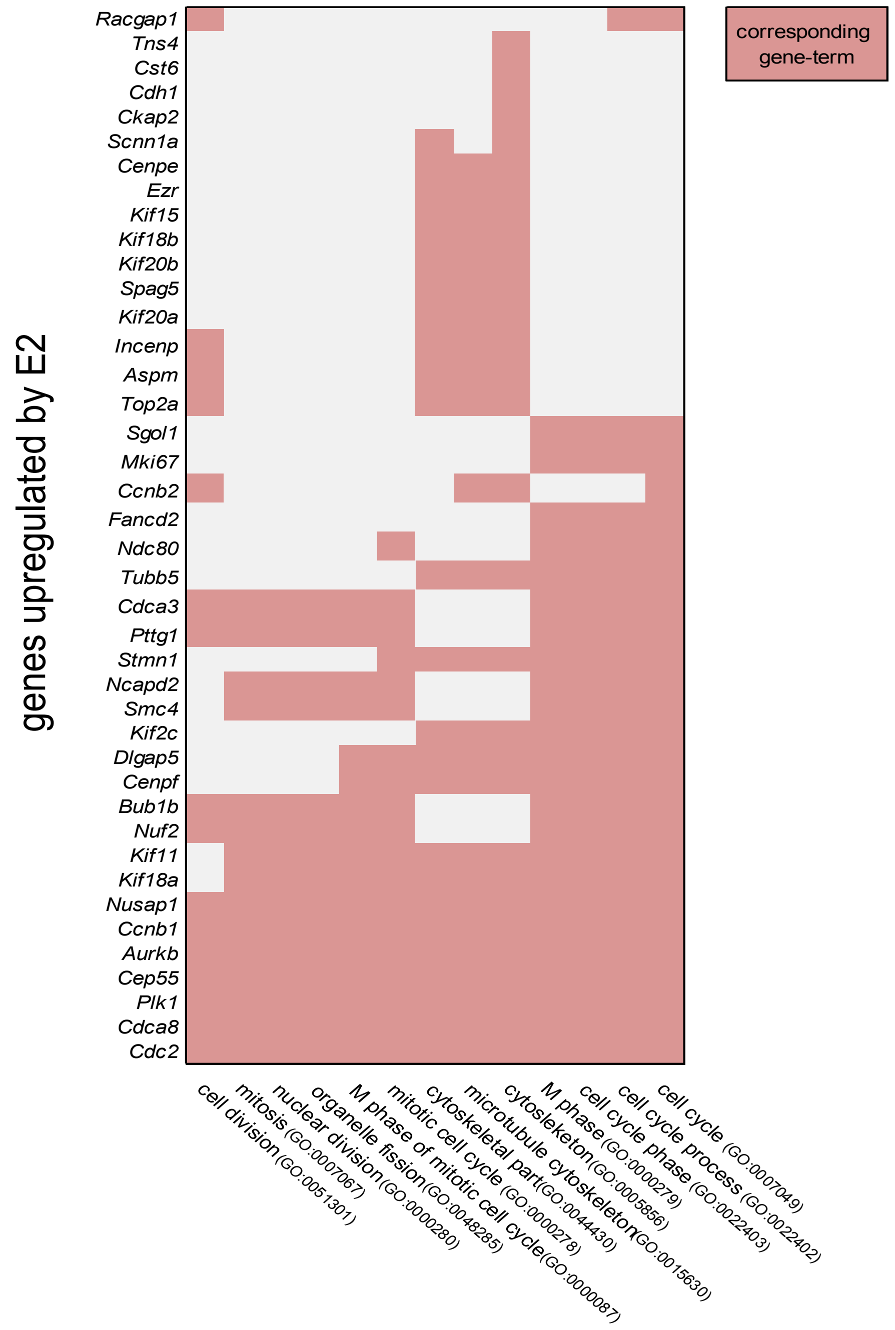

Figure S2. Functional clustering of E2-upregulated genes in the mammary gland. Functional annotation clustering was performed to assign E2-upregulated genes to associated biological processes (GO= gene ontology). P-value associated with each annotation (GO) term ≥ 0.01 as determined by EASE Score (modified Fisher Exact p-value).

References

Kanehisa, M., and S. Goto. 2000. “KEGG: Kyoto Encyclopedia of Genes and Genomes.” *Nucleic Acids Research* 28 (1): 27–30.

Kanehisa, Minoru, Susumu Goto, Yoko Sato, Masayuki Kawashima, Miho Furumichi, and Mao Tanabe. 2014. “Data, Information, Knowledge and Principle: Back to Metabolism in KEGG.” *Nucleic Acids Research* 42 (Database issue): D199–205. doi:10.1093/nar/gkt1076.
